# Comorbidity in trichotillomania (hair‐pulling disorder): A cluster analytical approach

**DOI:** 10.1002/brb3.1456

**Published:** 2019-11-06

**Authors:** Christine Lochner, Nancy J. Keuthen, Erin E. Curley, Esther S. Tung, Sarah A. Redden, Emily J. Ricketts, Christopher C. Bauer, Douglas W. Woods, Jon E. Grant, Dan J. Stein

**Affiliations:** ^1^ SA MRC Unit on Risk and Resilience in Mental Disorders Department of Psychiatry Stellenbosch University Cape Town South Africa; ^2^ Department of Psychiatry Massachusetts General Hospital and Harvard Medical School Boston MA USA; ^3^ Department of Psychology Temple University Philadelphia PA USA; ^4^ Department of Psychological and Brain Sciences Boston University Boston MA USA; ^5^ Department of Psychiatry and Behavioral Neuroscience University of Chicago Chicago IL USA; ^6^ Department of Psychology Florida State University Tallahassee FL USA; ^7^ Department of Psychiatry and Biobehavioral Sciences University of California Los Angeles CA USA; ^8^ Department of Psychology Marquette University Milwaukee WI USA; ^9^ SA MRC Unit on Risk & Resilience in Mental Disorders Department of Psychiatry and Neuroscience Institute University of Cape Town Cape Town South Africa

**Keywords:** borderline personality disorder, comorbidity, depression, treatment, trichotillomania

## Abstract

**Background:**

A promising approach to reducing the phenotypic heterogeneity of psychiatric disorders involves the identification of homogeneous subtypes. Careful study of comorbidity in obsessive‐compulsive disorder (OCD) contributed to the identification of the DSM‐5 subtype of OCD with tics. Here we investigated one of the largest available cohorts of clinically diagnosed trichotillomania (TTM) to determine whether subtyping TTM based on comorbidity would help delineate clinically meaningful subgroups.

**Methods:**

As part of an ongoing international collaboration, lifetime comorbidity data were collated from 304 adults with pathological hair‐pulling who fulfilled criteria for *DSM‐IV‐TR* or *DSM‐5* TTM. Cluster analysis (Ward's method) based on comorbidities was undertaken.

**Results:**

Three clusters were identified, namely Cluster 1: cases without any comorbidities (*n* = 63, 20.7%) labeled “simple TTM,” Cluster 2: cases with comorbid major depressive disorder only (*N* = 49, 16.12%) labeled “depressive TTM,” and Cluster 3: cases presenting with combinations of the investigated comorbidities (*N* = 192, 63.16%) labeled “complex TTM.” The clusters differed in terms of hair‐pulling severity (*F* = 3.75, *p* = .02; Kruskal–Wallis [KW] *p* < .01) and depression symptom severity (*F* = 5.07, *p *= <.01; KW *p* < .01), with cases with any comorbidity presenting with increased severity. Analysis of the temporal nature of these conditions in a subset suggested that TTM onset generally preceded major depressive disorder in (subsets of) Clusters 2 and 3.

**Conclusions:**

The findings here are useful in emphasizing that while many TTM patients present without comorbidity, depression is present in a substantial proportion of cases. In clinical practice, it is crucial to assess comorbidity, given the links demonstrated here between comorbidity and symptom severity. Additional research is needed to replicate these findings and to determine whether cluster membership based on comorbidity predicts response to treatment.

## Graphical abstract

Findings from analysis of data from one of the largest clinically diagnosed cohorts of trichotillomania (TTM) to date emphasize the prominence of depression in this condition, as well as an association between increased comorbidity and severity of hair‐pulling and depressive symptomatology. In the clinic, there should be cognizance of the potential presence of comorbidity and of depression in particular, that could impact on treatment choice, response, and outcome. In the presence of comorbidity, treatment for TTM may need to be modified.

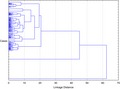

## 1 INTRODUCTION

Hair‐pulling disorder (or trichotillomania, TTM) is a psychiatric disorder characterized by chronic and problematic hair‐pulling, and classified in the obsessive‐compulsive and related disorders (OCRD) category of DSM‐5 and ICD‐11 (American Psychiatric Association, 2013). In these classification systems, the disorder is defined in terms of a single behavior, that is, hair‐pulling, but TTM may present with heterogeneous demographic and clinical features. As a result, several attempts have been made to delineate distinct hair‐pulling subtypes (Lochner, Seedat, & Stein, 2010) that are characterized by clinically meaningful profiles, underlying neurobiology, and treatment response. Posited subtypes include childhood‐onset TTM (Flessner, Lochner et al., 2010; Lochner et al., 2010; Odlaug, Chamberlain, Harvanko, & Grant, 2012), TTM with oral behaviors (Odlaug & Grant, 2008), TTM with self‐injurious behavior (Simeon et al., 1997), TTM with “focused” (i.e., with a compulsive quality) versus “automatic” (i.e., with decreased awareness) pulling styles (e.g., Flessner et al., 2008), and TTM subtypes positioned on opposite ends of the NEO‐FFI neuroticism/extraversion continuum (Keuthen, Tung, Tung, Curley, & Flessner, 2016). To date, however, no TTM subtype has had sufficient clinical utility to warrant inclusion in the official nosology.

Investigation of psychiatric comorbidity of TTM may assist in the identification of hair‐pulling subtypes. In clinical samples of patients with TTM, there are high rates of comorbid mood and anxiety disorders, and such comorbidity may be accompanied by more severe hair‐pulling, greater problems with resistance and control over hair‐pulling, poorer quality of life, and greater disability (Grant, Redden, Leppink, & Chamberlain, 2017; Grant, Redden, Medeiros, et al., 2017). Comorbidity with obsessive‐compulsive disorder (OCD) and with other body‐focused repetitive behaviors (BFRBs) such as skin‐picking disorder (SPD) is also common in individuals with TTM (Grant et al., 2016; Stein et al., 2008). In adults with TTM, co‐occurring OCD has been associated with greater anxiety and depression relative to TTM alone (Keuthen, Altenburger, & Pauls, 2014) and co‐occurring SPD has been associated with greater time spent pulling and picking, a greater likelihood of being triggered by visual cues, and trends toward greater impairment compared to TTM alone (Odlaug & Grant, 2008).

In OCD, the paradigmatic OCRD, examination of comorbidity contributed to the identification of tic‐related OCD and the specification of this subtype in DSM‐5 (Leckman et al., 1994). A cluster analysis of OCD found three distinct comorbidity profiles in OCD: a reward deficiency profile (i.e., trichotillomania, TS, pathological gambling, and hypersexual disorder), an impulsivity profile (i.e., compulsive shopping, kleptomania, eating disorders, self‐injury, and intermittent explosive disorder), and a somatic profile (i.e., body dysmorphic disorder and hypochondriasis), each of which were associated with different demographic factors and phenomenological qualities, such as age of illness onset and level of insight (Lochner et al., 2005). Clinically diagnosed samples of TTM have to date been relatively small, and cluster analysis has to date not been used to explore comorbidity in TTM. Multisite collaborations in TTM have recently provided samples with sufficient power to undertake more detailed analysis of comorbidity patterns in this disorder. In this study, our primary aim was to explore TTM subtyping by implementing a multivariate statistical technique (cluster analysis) (Skinner & Blashfield, 1982) to investigate psychiatric comorbidity in TTM. A second aim was to investigate the association of each of the identified clusters with specific demographic variables (i.e., age and sex) and clinical variables (i.e., onset and duration of illness, severity of depression symptoms, and TTM symptom severity).

## 2 METHODS AND MATERIALS

### 2.1 Subjects/sites

In a multisite international collaboration, lifetime comorbidity data were collated from individuals with pathological hair‐pulling, from two sites (one in the USA, and one in South Africa).

Individuals were recruited from studies investigating the genetic underpinnings of chronic hair‐pulling at Massachusetts General Hospital and Stellenbosch University between 2006 and 2015. Participants were recruited from multiple sources, including the TLC Foundation for Body‐Focused Repetitive Behaviors (BFRBs; i.e., a national advocacy organization for BFRBs), local mental health clinics, hospital intranets, and flyers posted in several relevant areas like hair salons and campuses. All subjects were between 18 and 73 years of age and met diagnostic criteria for *DSM‐IV‐TR* (American Psychiatric Association, 2000) or *DSM‐5* (American Psychiatric Association, 2013) for TTM.

Participant data were drawn from different clinical sites, some of whom have previously published comorbidity findings (Keuthen et al., 2014; Lochner et al., 2010). Comorbidity data from a subset of participants included in these earlier analyses were included in the present study. Data were typically obtained at an intake assessment and so comprise a broad range of patients, including those with current mood, anxiety, or substance use disorders, and with varying symptom severity and treatment history. Study exclusion criteria were mental retardation, psychosis, or an autism spectrum disorder.

### 2.2 Interview

Specific demographic data, including age when the participant joined the study and highest level of education, were obtained. To be included, participants met the Diagnostic and Statistical Manual of Mental Disorders (DSM‐IV TR or DSM‐5) criteria [1] for a primary diagnosis of TTM. The Structured Clinical Interview for Axis I Disorders (SCID‐I/P) (First, Spitzer, Gobbon, & Williams, 1998) was used to determine lifetime comorbidity.

Data were also collected on age of onset and severity of hair‐pulling and depressive symptomatology. Self‐report scales were used to assess the severity of hair‐pulling and depression, respectively. The Massachusetts General Hospital Hair Pulling Scale (MGHHPS; Keuthen et al., 1995) is a 7‐item measure of hair‐pulling severity used here, with items pertaining to frequency, intensity and control or hair‐pulling urges; frequency, intensity and control of hair‐pulling; and hair‐pulling‐related emotional distress. Items are rated on a 0‐ to 4‐point Likert scale with higher ratings indicative of greater hair‐pulling severity. Items are typically summed to yield an overall total score ranging from 0 to 28. MGHHPS demonstrates good internal consistency (Keuthen et al., 1995), excellent test–retest reliability, and fair to good convergent validity and discriminant validity (Diefenbach, Tolin, Crocetto, Maltby, & Hannan, 2005; O'Sullivan et al., 1995). The Beck Depression Inventory version I (BDI‐I) is a 21‐item self‐report rating inventory that was implemented to measure characteristic attitudes and symptoms of depression (Beck, Steer, & Garbin, 1988; Beck, Ward, Mendelson, Mock, & Erbaugh, 1961). Internal consistency for the BDI ranges from 0.73 to 0.92 with a mean of 0.86, depending on the nature of the cohort under investigation; for psychiatric populations, the internal consistency is high, with alpha coefficients of 0.86 (Beck et al., 1988).

### 2.3 Data analysis

Excel spreadsheets from two (2) sites with selected demographics and clinical data on comorbidity, age of onset and severity of TTM and depressive symptoms, respectively, were collated and cleaned. Duplicate cases were identified and removed, and cases without sufficient comorbidity data (i.e., at least some data on mood, anxiety, and obsessive‐compulsive related conditions) were removed. The final database of 304 cases was analyzed using Statistica software (StatSoft) (161 from MGH, and 143 from SU).

Cluster analysis (Ward's method), using Euclidean distance as the similarity measure, was conducted on all cases with sufficient data available on fourteen comorbidities. These were major depressive disorder (MDD), panic disorder with/out agoraphobia, social anxiety disorder (SAD), specific phobia, generalized anxiety disorder (GAD), OCD, SPD, posttraumatic stress disorder (PTSD), attention deficit disorder (ADD), tics, binge‐eating disorder, body dysmorphic disorder (BDD), alcohol use disorder (alcohol abuse and dependence), and substance use disorder (substance abuse and dependence). The association of identified clusters with demographic variables (age, gender) was determined using chi‐square analyses and one‐way ANOVAs as appropriate. To compare the differences in linear clinical variables (i.e., age of onset of TTM, duration of pulling, hair‐pulling severity, severity of symptoms of depression, and age of onset of comorbid MDD where relevant) among the clusters, one‐way ANOVAs with Bonferroni‐corrected post hoc analyses were performed. The level of significance was set at 0.05.

### 2.4 Ethics

At each of the respective sites, all participants provided written informed consent for study procedures after risks and benefits were explained, and for their data to be shared (anonymously) across study sites.

## 3 RESULTS

Data from 304 adult patients with TTM (287 women, 94.4%), with ages ranging between 18 and 73 years (mean: 32.8, *SD*: 12.1), were included in the analyses. In the total group, age of onset of pulling ranged from 1 year to 45 years (mean: 13.4, *SD*: 5.8), hair‐pulling severity scores (MGHHPS total score) from 1 to 26 (mean: 14.8, *SD*: 4.8), and depression rating scores (BDI‐I total score) ranging from 0 (no depression symptoms) to 36 (mean: 10.85, *SD* 8.43). A total of 168 participants of the total sample had lifetime MDD (55.3%) with data on age of MDD onset available for 46 (27.4%) of this subset (mean: 22.2 years, *SD* 10.4). In the total sample, the correlation between the MGHHPS and BDI‐I total scores was positive, but not statistically significant (*r* = .25, *p* = .06).

**Figure 1:**
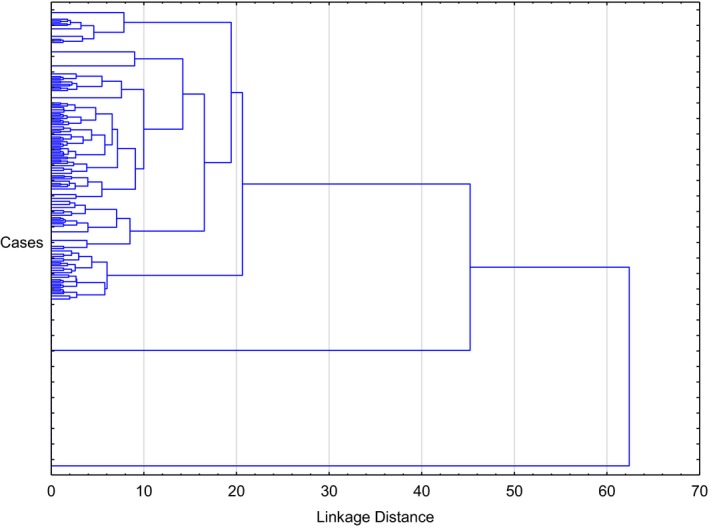
Dendrogram of 304 cases with trichotillomania (Ward's method, with Euclidean distances)

From the dendrogram of the cluster analysis (see Figure 1), three clusters were selected and labeled as follows: Cluster 1: “simple TTM” comprising of cases without any comorbidities (*n* = 63, 20.7%), Cluster 2: “depressive TTM” comprising of cases with MDD only (*N* = 49, 16.12%), and Cluster 3: “complex TTM” with cases presenting with combinations of the investigated comorbidities (*N* = 192, 63.16%).

The frequencies of each comorbid disorder per cluster are depicted by Table 1. Cluster 3 cases presented with one or more disorders in combination, with depression being most prevalent (in 62% of cases), followed by OCD (36%) and SPD (24%).

**Table 1:** Frequencies of each lifetime comorbid disorder, per cluster

Comorbid disorders	Cluster membership		
	Cluster 1: Simple TTM *n* = 63 *n* (%)	Cluster 2: Depressive TTM *N* = 49 *N* (%)	Cluster 3: Complex TTM *n* = 192 *n* (%)
Major depressive disorder	0	49 (100%)	119 (62%)
Panic disorder with/out agoraphobia	0	0	16 (8%)
Social anxiety disorder	0	0	28 (15%)
Specific phobia	0	0	43 (22%)
Generalized anxiety disorder	0	0	42 (22%)
Obsessive‐compulsive disorder	0	0	70 (36%)
Skin‐picking disorder	0	0	46 (24%)
Posttraumatic stress disorder	0	0	29 (15%)
Attention deficit disorder	0	0	15 (8%)
Tics	0	0	16 (8%)
Binge‐eating disorder	0	0	12 (6%)
Body dysmorphic disorder	0	0	17 (9%)
Alcohol abuse and dependence	0	0	26 (14%)
Substance abuse and dependence	0	0	43 (22%)

Abbreviation: TTM, trichotillomania.

In terms of demographic features, the three clusters did not differ significantly in terms of the sex distribution and current age. Clinically, age of onset of TTM and duration of illness also did not differ across the clusters (Table 2). Age of onset of MDD did not differ significantly across the relevant clusters (2 and 3). Age of onset of MDD data was available for a subset (*n* = 19) of Cluster 2 cases. In this cluster, the difference between the mean age of onset of TTM and MDD, respectively, was 9.5 (*SD* 10.9) years, with most cases presenting with TTM at a much earlier age (11 out of 19 cases, or 57.9%) or at the same age (three cases) as MDD. In Cluster 3, age of onset of MDD data was available for a subset of 27 cases. Here, the difference between the mean age of onset of TTM and MDD, respectively, was 6.4 (*SD* 8.8) years, also with most cases presenting with TTM at an earlier age (24 out of 27 cases, or 88.9%) or at the same age (two cases) as MDD. In this cluster, one case reported onset of MDD at an earlier age as TTM onset.

**Table 2:** Demographic and clinical characteristics across clusters of cases

Variable	Cluster membership			Statistic	*p*‐Value
	Cluster 1 Simple TTM *n* = 63	Cluster 2 Depressive TTM *N* = 49	Cluster 3 Complex TTM *n* = 192		
Sex					
Male	3	1	13	Chi‐square: 1.76	.42
Female	60	48	179		
Mean age (in years)	32.73 (*SD* 12.95)	33.37 (*SD* 11.77)	32.6 (*SD* 11.99)	*F* = 0.08	.93
Age of onset of TTM (in years)	12.92 (*SD* 5.45)	12.82 (*SD* 5.96)	13.65 (*SD* 5.92)	*F* = 0.61	.54
Age of onset of comorbid MDD^a^ (in years)	(no MDD)	23.4 (*SD* 10.5)	21.3 (*SD* 10.4)	*F* = 0.18	.50
Duration of illness (in years)	19.81 (*SD* 13.25)	20.55 (SD13.14)	18.87 (*SD* 12.17)	*F* = 0.4	.67
MGHHPS total score	13.38 (*SD* 4.28)	14.82 (*SD* 5.56)	15.27 (*SD* 4.65)	*F* = 3.75	.02*
BDI total score	3.7 (*SD* 3.16)	12.8 (*SD* 5.81)	12.4 (*SD* 8.76)	*F* = 5.07	.01**

Abbreviations: BDI, Beck Depression Inventory; MDD, major depressive disorder; MGHHPS, Massachusetts General Hospital Hair Pulling Scale; TTM, trichotillomania.

a Of the 168 cases with lifetime MDD, only 46 (27.4%) had age of onset of MDD data available.

* Cluster 3 cases had significantly worse hair‐pulling than those in Cluster 1 (*p* = .02).

** Clusters 2 (*p* = .04) and 3 (*p* = .003) both had significantly more severe depressive symptomatology than Cluster 1 cases.

**Figure 2:**
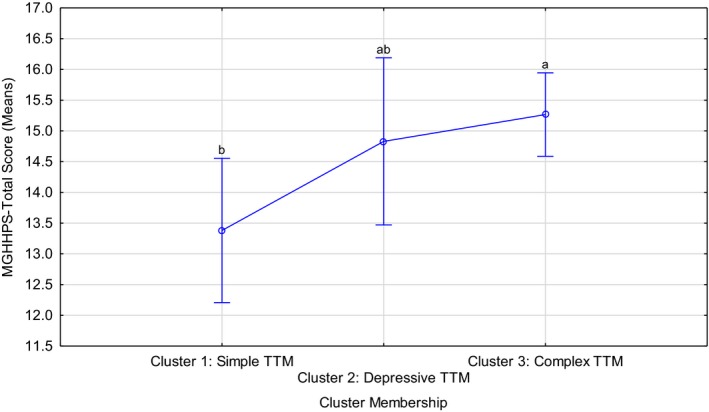
Comparison of trichotillomania (TTM) severity among the three identified clusters. Current effect: *F*(2, 295) = 3.75, *p* = .02, Kruskal–Wallis *p* < .01 Letters indicate post hoc differences at a 5% significance level, i.e. means without overlapping letters are significantly different

**Figure 3:**
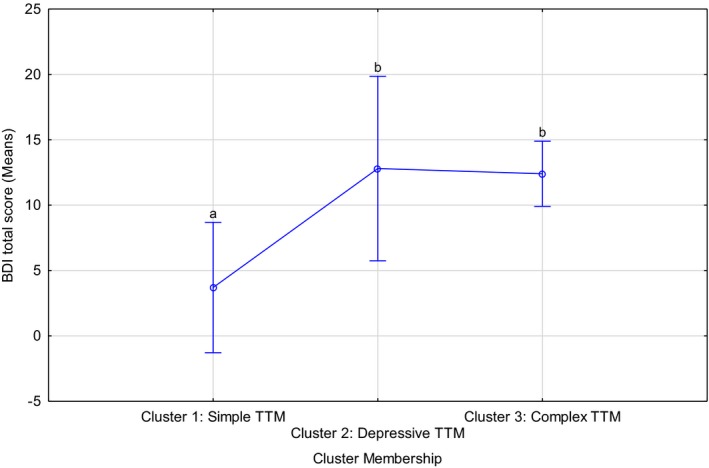
Comparison of severity of depressive symptoms among the three identified clusters. Current effect: *F*(2, 52) = 5.07, *p *= <.01 Kruskal–Wallis *p* < .01. TTM, trichotillomania; Letters indicate post hoc differences at a 5% significance level, i.e. means without overlapping letters are significantly different

The three clusters differed significantly in terms of hair‐pulling severity (*F*(2, 295) = 3.75, *p* = .02; Kruskal–Wallis [KW] *p* < .01) and depression (*F*(2, 52) = 5.07, *p *= <.01 KW *p* < .01), with an increase in the number of comorbid conditions being associated with more severe hair‐pulling and more severe depression symptoms (Table 2, Figures 2 and 3).

Post hoc analyses suggested that Cluster 3 cases had significantly worse hair‐pulling than those in Cluster 1 (*p* = .02, using Bonferroni corrections). In terms of the severity of depressive symptomatology, BDI data were available for only a subset of participants (55 out of 304, or 18.1%). In this subsample, participants in Clusters 2 (*p* = .04) and 3 (*p* = .003) both had significantly more severe depressive symptomatology than Cluster 1 cases (Figure 3). Hair‐pulling severity did not correlate with severity of depressive symptoms in Clusters 1 and 2, but there was a tendency for pulling and depression severity to be positively correlated in Cluster 3 cases (*r* = .3, *p* = .06).

## 4 DISCUSSION

In one of the largest clinically diagnosed cohorts of TTM to date, three distinct clusters of adult cases were identified based on lifetime comorbidity. The first cluster consisted of cases without any comorbidity and was labeled “simple TTM,” a second cluster of cases presented with comorbid depression only and was labeled “depressive TTM,” and a third cluster of cases with multiple comorbid disorders was identified and labeled “complex TTM.” The latter was the largest group (63% of the total sample), with most of its cases (62%) presenting with MDD in addition to one or more other conditions. There was a significant association between an increase in the number of comorbid disorders and hair‐pulling and depression severity, respectively.

The fact that the sine qua non of TTM is hair‐pulling has led some to suggest that hair‐pulling should be conceptualized as a symptom rather than a syndrome (O'Sullivan et al., 1997). Our finding that a large proportion of patients suffer predominantly from hair‐pulling and have no comorbid conditions indicates that TTM is not necessarily secondary to, or symptomatic of, another condition. Clinical experience with individuals with TTM shows that there are many syndromic aspects; e.g., hair‐pulling is triggered by similar cues, hair‐pulling has similar phenomenological aspects, and hair‐pulling has similar course, across different patients. The recognition of BFRB disorders in DSM‐5 and ICD‐11 is important in drawing attention to these under‐diagnosed conditions, and so increasing rates of treatment.

The finding that depression is a common comorbid disorder in TTM is consistent with previous findings that symptoms of depression are significantly higher in TTM participants than in healthy controls (Diefenbach et al., 2005; Duke, Bodzin, Tavares, Geffken, & Storch, 2009), with lifetime and current MDD occurring in between 29% and 52% of patients (Grant, Redden, Medeiros, et al., 2017; Houghton et al., 2016). Whereas Cluster 2 patients had comorbid MDD only and Cluster 3 patients had a range of different comorbidities. Notably, those with increased  comorbidity had more severe hair‐pulling symptoms. Previous authors have suggested that the direction of the relationship between TTM and depression is bi‐directional; that is, hair‐pulling and its sequelae may lead to depressed mood, and conversely depression may trigger pathological hair‐pulling as a way to regulate mood (e.g., Mansueto, Thomas, & Brice, 2007). Analysis of the temporal nature of these conditions in a subset suggested that TTM onset generally preceded MDD in (subsets of) Clusters 2 and 3. While our data cannot address causality, it may be argued that the successful treatment of TTM may reduce risk of developing depression. It may also be that untreated TTM in early life may be an indicator of future depressive symptoms. This relationship needs further exploration. The data rendered by our investigation ultimately emphasize the importance of screening for depression in TTM, and of also targeting depression symptoms for treatment.

OCD and SPD were the second and third most prevalent comorbidities in the Cluster 3 cases. OCD has previously been found to co‐occur in more than a quarter (Grant, Redden, Medeiros, et al., 2017) and SPD in almost a fifth (Keuthen, Curley, et al., 2016) of patients with TTM. It has been found that TTM cases with OCD have higher levels of anxiety and depression when compared with those without comorbid OCD. Cases with comorbid SPD also had increased grooming and greater functional impairment compared to those without SPD. In summary, the data here are consistent with previous work indicating that comorbidity of TTM, OCD, and SPD results in increased burden (Keuthen, Curley, et al., 2016), potentially placing individuals at risk for depression, and in greater need of modified treatment.

Further research is needed to determine whether cases in Clusters 2 and 3 require modifications of current treatment approaches. Modifications may entail more complicated (needing more experienced and specifically trained clinicians) and lengthier treatment during which comorbidities are also addressed. Although the evidence base for serotonin reuptake inhibitors (SRIs) in TTM is limited, it is possible that these agents are more useful when comorbid depression is present. More than a fifth of Cluster 3 cases also presented with GAD, specific phobia, or substance use disorders—disorders that may also be linked to depression, and which may also need clinical attention in themselves. More work is needed to determine whether hair‐pulling or comorbid disorders would require treatment first, or whether treatment of these should proceed in parallel.

In terms of study limitations, data on treatment history were available for a subset of participants only; future work would benefit from comprehensive collection of treatment response data. Similarly, data on severity of depressive symptomatology and age of onset of comorbid MDD were available for a subset only. It should also be noted that some statisticians have questioned the value of clustering methods to identify “naturally” occurring subgroups. Another possible limitation is that we have investigated a relatively limited number of lifetime comorbidities since the number of cases presenting with other comorbidities was too low to be added to the analyses. With increased sample size, this limitation may be addressed. Another possible limitation is that study participants may not be representative of TTM patients in the community. However, participants included here took part in genetics studies at both sites and were not drawn from specific treatment trials with additional exclusion criteria. Thus, the cohort included here was a *naturalistic* sample, likely representative of the TTM population in general.

## 5 CONCLUSIONS

In conclusion, findings from analysis of data from one of the largest clinically diagnosed cohorts of TTM to date emphasize the prominence of depression in this condition, as well as an association between increased comorbidity and severity of hair‐pulling and depressive symptomatology. In the clinic, there should be cognizance of the potential presence of comorbidity, and of depression in particular, that could impact on treatment choice, response, and outcome. In the presence of comorbidity, treatment for TTM may need to be modified. Further work is warranted to replicate these findings and to determine whether cluster membership based on comorbidity profile can predict treatment response and outcome. Finally, the extent to which the identified clusters correspond with previously identified subtypes (e.g., pulling style or personality types) needs further exploration to ensure that we are building a cumulative understanding of TTM subtyping.

## CONFLICT OF INTEREST

None declared.

## Data Availability

The data that support the findings of this study are available from the corresponding author upon reasonable request.
